# A patient-centered interactive voice response system for supporting self-management in kidney transplantation: design and field testing

**DOI:** 10.3389/fdgth.2024.1386012

**Published:** 2025-02-07

**Authors:** Raheleh Ganjali, Mahin Ghorban Sabbagh, Saeid Eslami

**Affiliations:** ^1^ Clinical Research Development Unit, Emam Reza Hospital, Mashhad University of Medical Sciences, Mashhad, Iran; ^2^ Department of Medical Informatics, Faculty of Medicine, Mashhad University of Medical Sciences, Mashhad, Iran; ^3^ Kidney Transplantation Complications Research Center, Faculty of Medicine, Mashhad University of Medical Sciences, Mashhad, Iran; ^4^ Department of Nephrology, Faculty of Medicine, Mashhad University of Medical Sciences, Mashhad, Iran; ^5^ Pharmaceutical Research Center, School of Pharmacy, Mashhad University of Medical Sciences, Mashhad, Iran

**Keywords:** user-centered design, self-management, interactive voice response system, kidney transplant, usability, CeHRes roadmap

## Abstract

**Introduction:**

Self-management is the ability to control one's own responses to treatments, physical and psychological side effects, and lifestyle choices related to a chronic condition.

**Purpose:**

To describe the development of a standard and practical user-centered design process for an interactive voice response system (IVRS) to improve self-management in kidney transplant (KT) recipients.

**Methods:**

The IVRS was constructed utilizing the four phases of the Center for eHealth and Wellbeing Research (CeHRes) roadmap: the contextual inquiry, the value specification, the design phase, and evaluation. First, a literature review, background analysis, and needs assessment were used to identify the needs and problems and solutions related to self-management of KT recipients. Then, with the help of a team of experts and KT recipients, a logic model was created and evaluated. The IVRS was developed through iterative design development in response to these findings. Finally, fifteen end users (KT beneficiaries and health professionals) participated in a usability field test by completing a thinking -aloud test and a questionnaire based on the System Usability Scale (SUS).

**Results:**

The review study indicates the necessary of self-management education and the potential outcomes and functionalities of information technology intervention. The situation analysis and needs assessment led to the final important requirements for the design of the intervention. All values were identified in three meetings with principal stakeholders, and a logic model was designed. The user test yielded an average SUS score of 81.2, and these results served as the basis for the usability requirements. Health Care Providers (HCPs) struggled with storing the profile of registered patients, setting up medication and personalizing adherence calls, and educational calls and follow-ups.

**Conclusion:**

Following the CeHRes roadmap, an intervention based on IVRS was developed with considering the needs and preferences of KT recipients and HCPs. Designers and researchers could use the CeHRes roadmap as a reference when developing IT-based intervention systems. However, decisions must be made about the thoroughness of the execution of each phase, taking into account time constraints.

## 1 Introduction

Kidney transplantation (KT) is the transfer of a healthy kidney from a matched donor into the body of another person with renal failure (1). It is the most promising choice for patients with end-stage renal disease (2). After KT, patient's compliance to a comprehensive and continuous regimen of medical advice plays a crucial role in determining both short and long-term results (3). Prior studies have demonstrated that non-adherence to immunosuppressants is linked to a 60% higher risk of graft rejection among KT recipients (4). Several observational studies have reported elevated rates of non-adherence to lifestyle recommendations (5, 6). For instance, Kobus et al. revealed that 85.3% of patients did not alter their diet post-kidney transplant, while 64.2% were unaware of dietary guidelines (7).

Self-management is managing symptoms, treatments, psychological and physical complications, and lifestyle behaviours associated with a chronic disease (8). Currently, self-management is considered a significant aspect of successful health care. It significantly improves patients' health status and quality of life, however reduces the rate of hospital readmissions (9). Inadequate self-management may result in rejection of a graft (9).

Automated telephone intervention approaches may be necessary to overcome the various barriers to improving self-management. Interactive voice response system (IVRS) allow users to engage with a series of structured voice-recorded messages and can provide responses to inquiries using their touch-tone phones (10). IVRS has many advantages over other digital health solutions (11). IVRS calls can be more time saving than other self-monitoring methods, which can be especially helpful for people with low literacy skills (12, 13). Listening to voice prompts and responding with simple numeric answers can be much less cognitively and numerically demanding than producing a detailed report of self-monitoring data (14). IVRS includes facilitating two-way real-time communication, such as soliciting inquiries and receiving responses, as well as personalized interventions. Given the relatively low per-contact cost of IVRS, regular health monitoring messages can be dispatched to maintain communication with patients. Furthermore, due to the ubiquitous nature of mobile phones carried by patients, instances of missed or unsuccessful calls are highly improbable. IVRS can also be used to provide timely feedback in response to self-monitoring data. A review of studies has found that IVR for self-monitoring leads to promote clinical outcome (15). This study has showed IVRS interventions lead to alternation health behaviours of patients, and enhance healthcare utilization, yielding positive impacts in various crucial domains such as immunization, screening, appointment attendance, and adherence to medications or tests (15). Another review study has shown IVR-based interventions exhibit promise in influencing specific health behaviours, notably medication adherence and engagement in physical activities (16). IVRS has been applied in many healthcare settings. Some studies have investigated the effectiveness of the IVRS in reducing cardiovascular risk in metabolic syndrome (17) and other studies have investigated effectiveness in physical activity (13, 18). This system has been effective in evaluation of adverse events after discharge of emergency department (19).

Digital health products are acknowledged as necessary for the sustainability of healthcare systems, and the quality of software, particularly usability, plays a vital role in their success and acceptance (20). A fundamental strategy for the development of digital solutions is usability testing, a widely employed method to assess whether designated users can effectively and efficiently achieve the intended use (21). A digital product success in large scale depends on the presence of sufficient usability (22). Usability in digital health products can have a significant impact on patient care; enhanced usability can result in more efficient completion of tasks, reduced errors, and improved treatment outcomes (20). There are many studies focused on the usability of IVRS (14, 18, 23), but there are few studies on the usability of IVRS for self-management and in patient populations. This study attempts to fill this gap by reporting on our development method and evaluating IVRS usability. Thus, the aim of this study is to develop an ordinary and practical user-centred design process for an interactive voice response (IVR) system to improve the self-management of KT recipients (especially for patient education and monitoring). We assume that all participants contributing to this usability testing study of the IVRS will provide valuable suggestions for further advancement.

## 2 Methods

**Figure 1:**
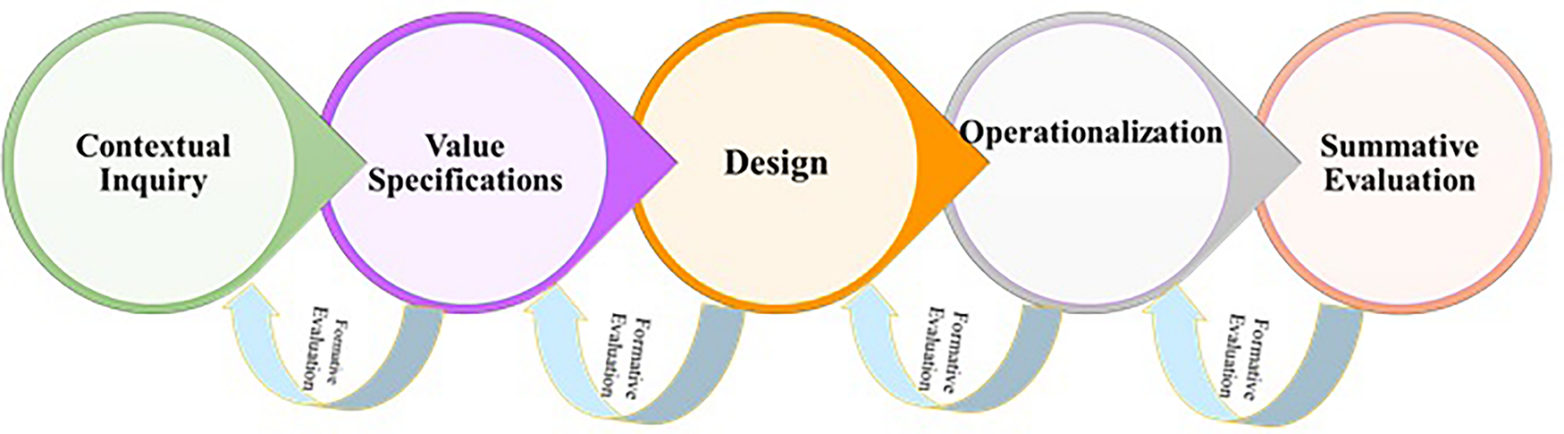
Cehres roadmap.

The IVRS was developed by using the Center for eHealth and Wellbeing Research (CeHRes) roadmap and User Centered Design (UCD) process. The CeHRes roadmap outlines a practical methodology to provide guidance to practitioners (such as designers, developers, and project managers) and researchers in the development and implementation of eHealth innovations. As shown in Figure 1, CeHRes roadmap includes five distinct stages for investigating and assessing the appropriateness of an eHealth technology for the intended audience and for its effective implementation in real-world settings (24).

**Figure 2:**
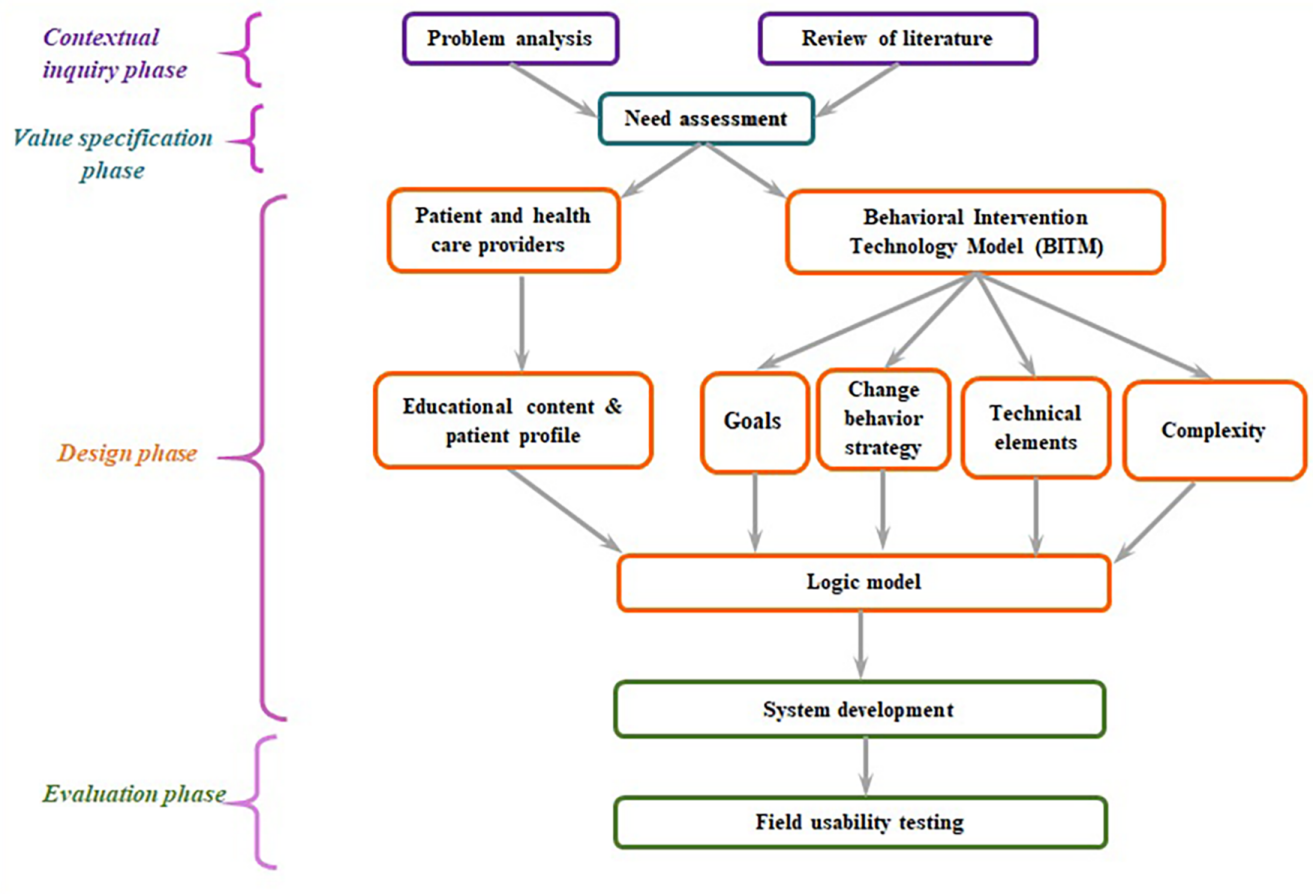
Cehres roadmap for designing of IVRS.

The CeHRes roadmap in this study includes four phases: (1) contextual inquiry phase (literature review and background & problem analysis) (2) value specification phase (needs analysis), (3) design phase (logic model and content), and (4) evaluation phase (usability and field testing) (Figure 2).

The UCD methodology allows for direct end-user involvement throughout the design process and supports the development of a tool that produces the best results (25). This technique involves rigorous usability testing of the initial release to find areas where feature and usability improvements are needed (26). This method is highly iterative in and of itself. Phases were repeated to evaluate intermediate designs, and to improve system as well as optimize the system or application (27).

Involving end users in the development and evaluation process helps researchers and developers design acceptable, practical, and validated tools that are appropriate for the end-user context. A user-based approach allows researchers to solicit feedback from patients and providers.

### 2.1 Contextual inquiry phase

This phase contains studies that investigate KT recipient's needs and preferences.

#### 2.1.1 Review of literature

This process takes place in two phases. In the first section, we conducted a systematic review to assess how Information Technology (IT) tools affect KT recipients' ability to self-manage. The PICO in this study included: KT recipients population, IT based intervention and self-management outcomes. All related keywords about above concepts was extracted and searched.

#### 2.1.2 Background and problem analysis

To identify patients who were non-adherent to immunosuppression among 230 renal transplant patients, we used the Basel Assessment of Adherence to Immunosuppressive Medications Scale (BAASIS) for reviewing medication adherence rate. Then, KT recipients completed Immunosuppressive Therapy Barriers Scale (ITBS) questionnaire to recognize the barriers of adherence to immunosuppressive medications.

### 2.2 Value specification phase

Patients' needs in terms of self-management plans were identified in the previous phase, as they arose naturally from patients' perceived problems. An additional needs assessment was conducted to further explore the specific needs and treatment requirements associated with using of self-management programs (28). To develop self-management interventions with optimal usability and feasibility, a deep and early understanding of the perspectives of both patients and the medical community was considered essential (29). Therefore, a study involving focus group interviews with both KT recipients (*n* = 12) and HCPs (*n* = 8) was conducted to (1) determine their readiness to use IVRS for self-management and (2) explore needs and preferences regarding the content of the intervention (30).

### 2.3 Design phase

Two objectives were established for this phase (Figure 2, Phase 2); determine the functionalities of the system and design the process, and content of the intervention. In this step, the target behavior was determined. The components of the intervention were designed by using the Behavioral Intervention Technology model (BIT) (Table 1) (31).

**Table 1:** BIT elements for IVRS.

BIT questions	BIT elements	IVRS elements
Why intervention?	Clinical and functional objectives	Improve medication adherence, quality of life, re-hospitalization rate, follow up visit
How is behavior change achieved?	Behavior change strategies	Education, feedback, Increasing motivation, self-regulation theory, Reinforcement
What technical elements do you use?	Elements	Providing information, reminders, logs, reports
How and when can it be provided technically?	Complexity	Personalized information and reminder
	Work flow	Time-based condition, performance-based condition, event-based condition

#### 2.3.1 Technical model

First, a behavioral analysis was conducted based on former phase to determine what should be changed in terms of the patient's abilities, opportunities, and motivations. Second, the collaborative method was used to form an expert panel of HCP involved in KT care and KT recipients to determine the dimensions of self-management improvement (32). The expert panel consisted of nephrologists, patient education specialists, nurses, and medical informaticians. Third, the expert panel agreed on feasible intervention components, behavior change techniques (BCTs), appropriate users, and circumstances. Logical modeling was used to present the potential mechanism of action of the intervention, along with any supporting information and hypotheses that illustrated the relationship between the immediate and long-term effects of the intervention on outcomes (33).

#### 2.3.2 Educational content and patient profile

To determine what HPCs expected from KT grantees, the expert panel conducted three meeting focus groups with a total of 10 people. At these meetings, an educational manual for transplant patients was assessed, and educational materials were revised and validated according to the consensus of the HCPs and the needs assessment. In three rounds of Delphi, the expert panels identified the information needed for patient profiling.

#### 2.3.3 System development

In order to create, schedule, receive, enter, and record calls, several technologies were integrated into the IVRS. We used the Linux Cent operating system, PHP for programming, a VoIP network, and a MySQL server connected to the Asterisk server to create the IVR call flow/script.

### 2.4 Evaluation phase

#### 2.4.1 Field usability testing

All functions of the IVRS were assessed by KT recipients, while the functions that depended on the role of HCP were appraised by the HCPs. During this phase, the IVRS underwent usability tests in the field, wherein a high-fidelity prototype was employed within the actual context where the intervention would be implemented. These tests were conducted with KT recipients (*n* = 10) and HCPs (*n* = 5), using the thinking-aloud method (34, 35), as suggested by Nielsen (27, 36). Task completion, user problems, and satisfaction were the three quality factors examined in this study.

Participants were observed working with the IVRS, and two researchers asked them to think aloud to clarify their decision making and to express user difficulties and errors encountered (37). Two evaluators managed the testing sessions and executed a thorough analysis of the findings. The researchers employed the verbal protocol methodology to gather data. Despite the verbal protocol being the traditional method with restricted probing techniques in relation to more interactive user engagement approaches like communication-based and coaching methods, it proficiently replicates a true contextual experience by refraining from rendering any external support to the participants (38, 39). The participants were directed to think aloud (verbalization their thoughts) while engaging in problem-solving tasks, with an emphasis on the notion that the intent of these activities was not to assess their digital competencies but rather to evaluate the usability of IVRS. Additionally, field notes were meticulously recorded throughout the problem-solving endeavors to document any observed technical challenges, user-friendliness, and learning processes, as well as nonverbal behaviors pertinent to task management. The evaluator prompted the participants to maintain their verbalization when they ceased doing so. In instances where a participant struggled to resolve a task after multiple attempts, the evaluator offered a cue to ascertain whether and how the task could be resolved. The observational lists were argued by the researchers to achieve a consensus regarding task performance and the recognized issues and errors. Following task analysis, patients completed the validated 10-item System Usability Scale (SUS) to get a comprehensive picture of usability (40). Each item was assigned a 5-point Likert rating, and the scores were summed to obtain a total score (the range is 0–100; a score of at least 70 is considered appropriate) (40, 41). The IVRS possible to assess the relevant functions for HCPs. In order to evaluate the severity of each problem encountered, a score ranging from 0 (indicating no usability problem) to 4 (indicating a usability catastrophe) was assigned (42). Tasks were classified as successfully completed (1 point), partially completed (0.5 points), or not successfully completed (0 points) (41).

The KT recipients' tasks included the following.

• Task 1: Call to IVRS and receive information about immunosuppressant drug
• Task 2: Answer the immunosuppressive adherence calls of IVRS and receive a 0 feedback message
• Task 3: Answer the informational calls.
• The HCPs tasks included the following:
• Task 1: Registered and insert patient profile
• Task 2: Setup individualized and grouping adherence and educational calls
• Task 3: Review reports of medication adherence and related feedback
• This study was performed in outpatient post-transplant clinic belonged to the Montaserieh Hospital Transplant Center in northeast of Iran.

## 3 Results

### 3.1 Contextual inquiry phase

The results of review study showed that the most of the studies (approximately 50% for clinical outcomes and 88.8% for study outcomes) had statistically significant effects. The knowledge about self-management subject, medication adherence, quality of life, unplanned admission and follow up visit were the most frequent outcomes in studies respectively. The media used were smartphones, wearable technologies, computer systems, and multicomponent systems. Technology functionalities were inform, instruct, remind and communicate in these studies. Information technology is increasingly used to inform patients and provide better treatment options for various diseases. The full results of the study have been published (27).

Based on ITBS, the frequently barriers stated by KT recipients included: taking many tablets of immunosuppressive medications at the same time, misconceptions about the usefulness of immunosuppressive medications, confusion about how to take medications, and difficulty remembering to take medications. The full results of the study have been published (43).

### 3.2 Value specification phase

An analysis of our needs led to an overview of the potential benefits and barriers associated with using an IVRS to support self-management and early content ideas intervention. Both patients and HCPs have emphasized the need for multicomponent and individualized education to improve patients' self-management skills by assessing health status and providing appropriate information, decision support, and feedback on recommended behaviors. Interventions should complement regular contact with HCPs and provide proper self-management support from HCPs. Both patients and HCPs expressed doubts about (real-time) monitoring of symptoms due to time constraints. In addition, interventions should be engaging, open, challenging, and safe. Finally, patients emphasized that IVRS use should be a choice and never forced. Based on these findings, requirements for intervention design were formulated. HCPs need to a dashboards for reviewing patient self-management behaviors and taking immunosuppressive drugs.

### 3.3 Design phase

#### 3.3.1 Educational content and patient profile

The educational material was divided into the following seven sections: (1) Sexual Activities and Pregnancy, (2) Infections and Methods of Prevention, (3) The Immune System and Its Role in Rejection, (4) Nutrition, (5) Long-Term Care, and (6) Return to Work and Life. There were two to eight subsections in each section.

The final profile of the patient was divided into five main areas, including; (1) demographic data (unique number, age, sex, education level, cell phone number, city, type of residence, and address of the patient), (2) type of immunosuppressive drug and dosing interval, (3) medical details (date of transplant, donor), (4) passcode of the patient, and (5) follow-up of the patient and date of referral.

#### 3.3.2 Recording vocal messages

The process involved converting educational material into concise vocal messages which were then recorded with utmost clarity. These messages were subsequently organized and classified into various subject areas. Voice menus were then created and implemented to facilitate input calls. KT recipients who dialed 31806 were directed by the system to access their desired voice messages. In order to cater to individual preferences and requirements, the output calls and reminders of IVRS were tailored using the patient's profile and their specific requests and needs.

#### 3.3.3 Logic model

**Figure 3:**
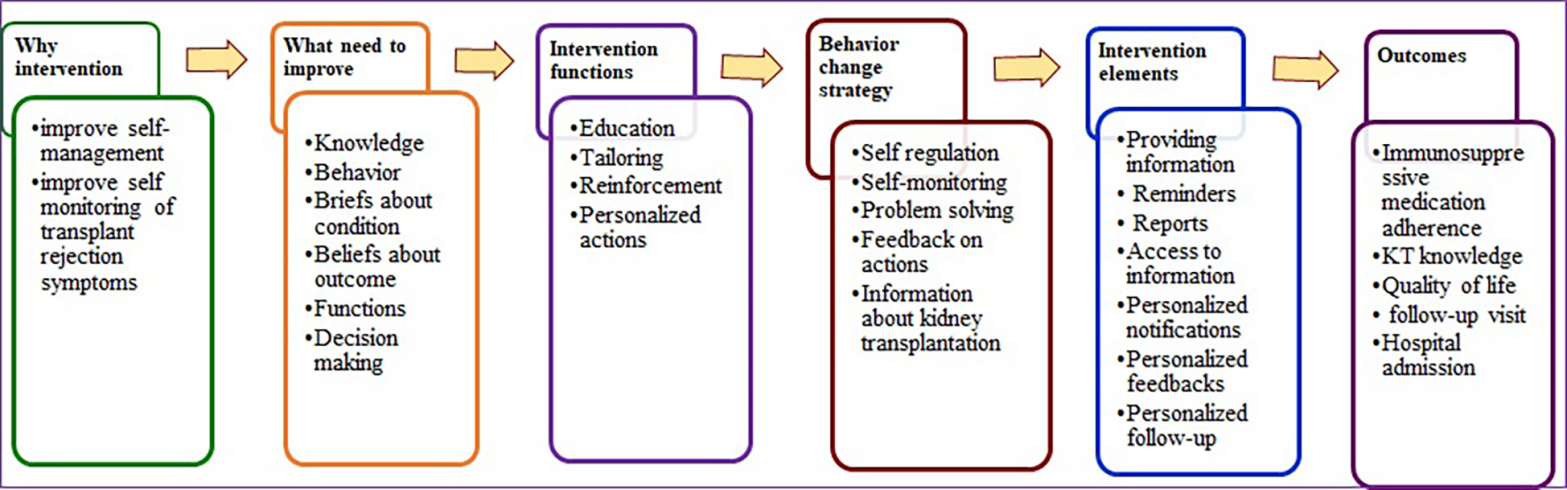
Logic model of IVRS intervention to improve self-management in KT recipients.

During the design phase, the study team initially focused on the two target behaviors: (1) improved access to tailored self-management activities and early detection of rejection, and (2) self-monitoring of symptoms. These behaviors were anticipated to be the most feasible, offered the opportunity for progress, and contributed in reducing rejection. Figure 3 shows the logic model of the intervention, which incorporated all of the evidence gathered in Phases 1 and 2, as well as the decision to use the final intervention features and BCTs.

#### 3.3.4 Development IVRS

**Figure 4:**
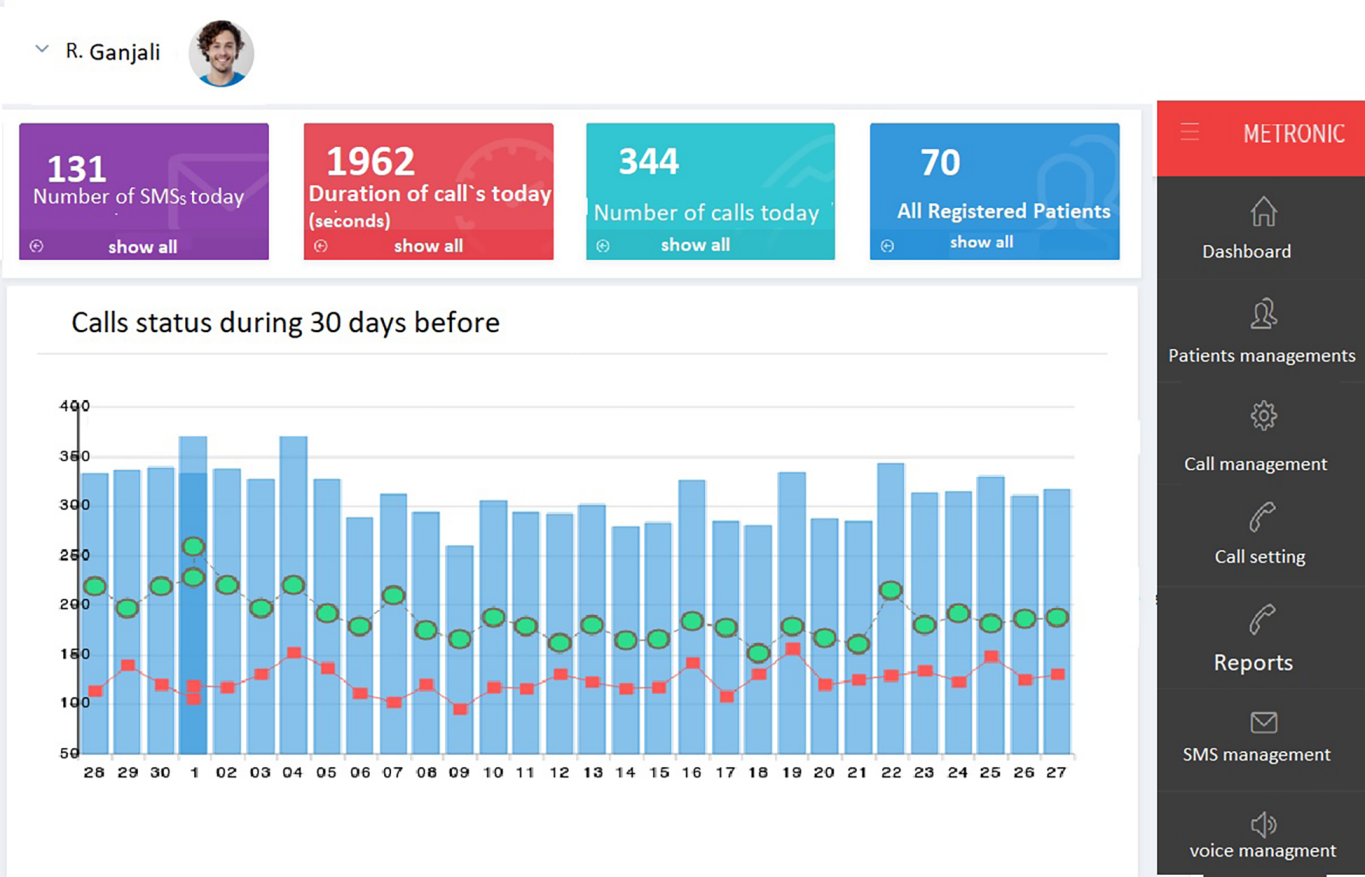
A screenshot of developed IVRS; this chart shows the status of calls over the last 30 days, with the blue bars showing the number of all calls that day, the green nodes showing the number of completed calls, and the red nodes showing the number of missed calls. The main menu on the left side of the figure allows the IVRS administrator to navigate to other pages in the application.

This system consisted three types of calls, consisted of educational, medication adherence and immunosuppressive drugs reminders. For each patient, all profile information and calls setting must be entered. Educational calls included educational materials and were conducted once every three days. Immunosuppressive calls was set based on the most important immunosuppressive drugs, while medication adherence calls were asked questions about use of daily drug. Based on these calls, the system was created a feedback as text message to support patient self-management. These contained a motivational message based on the patient's medication taking over the past week. In addition, every interaction the system with the patient is recorded in the patient profile as patient's reports. The patient's reports also include the date, time, and content of short message services (SMS) and calls. Figure 4 shows a screenshot of the web application used to manage IVRS.

### 3.4 Evaluation phase

IVRS has input and output calls. Input calls refer to calls that patient contact to system and follow the voice messages for receiving their answers, and output calls included all contacts of system to patients automatically. Output calls were considered as input calls for patients. In this study, input calls evaluated by patients and output calls evaluated by HCPs.

#### 3.4.1 Patients: task completion, problems and satisfaction

All ten users (100%) completed tasks 2–3, and 100% of subjects and about half of them successfully completed task 1. There was only one usability issue that occurred in Task 1: the subsections of each section were too long. The thinking aloud test did not reveal any new usability issues. The question about overall satisfaction with the tasks showed that all ten participants with tasks 1, 2, and 3 were most satisfied with the IVRS (average rating of 1). The results of the total value in Table 2 were calculated using the SUS (System Usability Scale). The percentage score was calculated based on the total respondent score for each question and the maximum total respondent score. The adjectival score for IVRS reached 81.2 (excellent).

**Table 2:** Mean rating of the SUS items of all participant (scale from 1—awful to 5—excellent).

List of Questions	1	2	3	4	5	Mean	SD
I think this system is easy to use	0	1	1	3	5	4.2	1.03
I found this system is not very complicated	0	1	1	4	4	4.1	0.99
I think I will use this SYSTEM	1	1	1	4	3	3.7	0.99
I think I don't need assistant to use this system	1	2	1	2	4	3.6	1.62
I find the various functions in this system were integrated	1	1	1	3	4	3.8	1.40
I think the contents of this system are quite consistent	1	0	1	4	4	4	1.25
I have no difficulty using this system	0	1	2	1	6	4.2	1.37
I feel comfortable using this system	0	1	1	3	5	4.2	1.03
I think the quality of the information in the system is very useful	0	0	1	3	6	4.5	0.71
I think that I would like to use this system frequently	0	1	1	2	6	4.3	1.06

#### 3.4.2 HCPs: task completion, problems and satisfaction

Of the 15 tasks executed by the five HCPs (with each user undertaking three tasks), a total of 10 tasks (66%) were successfully accomplished, while 5 tasks (34%) were not completed. In total, 18 usability issues were identified during this evaluation phase; however, 9 issues persisted following the removal of duplicate instances. Table 3 show the issues expressed by the users along with their associated tasks.

**Table 3:** The problems recognized by HCPs in usability evaluation.

Number	Problems	Number of user	Related task	Severity
1	Alerts pertaining to data that remains unrecorded, which must have been documented, are issued with a considerable delay.	4	1	4
2	The arrangement of specific components within the user interface, especially the icons, demonstrates a lack of clarity	4	3	4
3	There is an absence of instructions to facilitate the execution of a step when required by the user	4	2,3	3
4	The symbols inadequately convey their intended functions	3	2	3
5	The menus and some tab lack clear and appropriate labels	2	3	2
6	The heading designed to filter the reports is not perceivable by the users.	3	3	1
7	Actions are not readily accessible, as users are compelled to scroll down the screen.	1	3	1
8	The small font size poses difficulties for users when interpreting the reports and visual data presentations	2	4	0
9	The coloration of buttons fails to adequately convey their functional purposes	1	3	0

##### 3.4.2.1 Task completion

The degree to which a user can effectively and accurately fulfill objectives of performing tasks, referred to as effectiveness, was quantified by the ratio of successfully completed tasks to the overall number of tasks undertaken.The findings indicate that Task 1 was executed by all participants (100%). The participants attributed their complete execution of this task to its simplicity, and the absence of the need to anticipate subsequent steps. Conversely, Task 2 was not entirely completed by all users (60%). The participants cited the necessity of conducting the task across two distinct sections of the system as the reason for its incomplete execution. Similarly, Task 3 was also not fully completed by users (60%). The stated reasons for this included the ambiguous functionality of the items presented on the page and the absence of assistance in this section of the system.

##### 3.4.2.2 User problems

In HCP’ use of the app, 9 user errors and problems were identified based on task analysis and observations made during random navigation of IVRS. HCPs experienced problems with the management of patients' profiles. Another serious problem was related to setting up medication adherence details (severity level 4). Two less severe problems were related to personalization of information calls and the follow-up visit (severity 3). The lowest severity levels (0–2) were assigned to five problems, reporting medication adherence chart and feedback messages, setting scenario for calls.

##### 3.4.2.3 Satisfaction

The average of HCPs satisfaction core was 88.8 (SD 2.4) (Table 4). All HCPs reported the usefulness of all functionalities and the use of the system. However, most of HCPs reported that different functions for supporting self-management integrated in system and they like to use system frequently. Based on SUS rating, this system was excellent and acceptable with grade B (44).

**Table 4:** Mean rating of the SUS items of all HCPs (*n* = 5) (scale from 1—awful to 5—excellent).

List of Questions	1	2	3	4	5	Mean	SD
I think this system is easy to use	0	0	1	1	3	4.4	1.2
I found this system is not very complicated	0	0	3	0	2	3.8	1.4
I think I will use this SYSTEM	0	0	0	0	5	5	2.2
I think I don't need assistant to use this system	0	0	2	3	0	3.6	1.4
I find the various functions in this system were integrated	0	0	0	1	4	4.8	1.7
I think the contents of this system are quite consistent with process care	0	0	0	2	3	4.6	1.4
I think that I could support patients via system	0	0	0	3	2	4.4	1.4
I feel comfortable using this system	0	0	1	3	1	4	1.2
I think the system functionalities is very useful	0	0	0	0	5	5	2.2
I think that I would like to use this system frequently	0	0	0	1	4	4.8	1.7

## 4 Discussion

Personalized lifestyle recommendations and the self-management strategies can assist patients in altering their health behaviors and enhancing their functional capabilities. Digital tools possess significant potential in bolstering patient self-management, attributed to their efficacy, cost-effectiveness, continuous availability, and the provision of automated feedback. In this context, we formulated a comprehensive, eHealth-supported educational care pathway for individuals who have undergone kidney transplantation, adhering to the CeHRes framework and employing a literature review alongside eHealth focus groups with healthcare professionals (HCPs) and a patient advisory panel. The objective of the care pathway is to empower KT recipients to effectively manage their condition by furnishing them with immediate feedback regarding their personal health behaviors, particularly in relation to medication adherence. This process ensures that the user, with his or her wants, needs, and requirements, is always at the center of the development process of the IVRS. The UCD approach to system development improves functionality, usability, and the likelihood of intervention effectiveness (44). Both HCPs and patients perceived the concept of the Interactive Voice Response System (IVRS) as feasible, acceptable, and beneficial. The primary advantages of the IVRS were deemed to include the integration of real-time feedback on individual behaviors, the emphasis on goal-setting, and the activation of patient engagement.

### 4.1 Design process

The contribution by the patients in the expert panel resulted in a final version of IVRS that enables KT recipients to view the results of their self-management in an accessible, appealing and intuitive way. This study involved the co-design-driven development of IVRS, a digital self-management tool for use in KT process.

Studies indicates that barriers to adherence often stem from forgetfulness, lack of knowledge, and complex medication regimens (45, 46). The integration of the ITBS and e-health solutions, including reminders, plays a crucial role in enhancing adherence to immunosuppressive therapy (IST) among KT recipients. E-health interventions, such as electronic reminders, can effectively address these barriers by providing timely notifications and educational resources (47). E-health platforms can provide tailored information about medication regimens, enhancing patient knowledge and confidence (48).

In the design process of an eHealth-based intervention, it is imperative to draw upon theoretical frameworks, evaluate the most contemporary evidence, assimilate contextual information pertaining to the setting in which the intervention or application will be implemented, and engage all principal stakeholders—particularly patients (49, 50). In the present investigation, the Interactive Voice Response System (IVRS) was constructed upon a foundation of several behavioral change theories.

Feedback increase the efficacy of e- health interventions by promoting user comprehension and engagement, which are essential components for informed decision-making, behavioral modification, and self-monitoring outcomes (51, 52). Behavioral interventions, which include counseling, reminders, self-monitoring, and feedback regarding medication adherence, have demonstrated substantial advancements in medication compliance (4, 53–56). A notable function that was considered involved the provision of feedback concerning the intake of immunosuppressive medications.

Personalization is necessary to ensure that the system is useful for users, and different characteristics such as country of origin, gender, age, or comfort with the technology should be taken into account. A person-centered approach in the development of new health technology systems is essential to ensure that applications can be better tailored to the needs of different ageing populations (57). Personalization is frequently cited as one of the important advantages of e-mental health applications and is also related to increased engagement (58, 59).

Many studies have been applied CeHRes Roadmap to design and development digital health interventions (48, 49, 58, 60–63). By incorporating the diverse groups of stakeholders at each relevant phase, we have ascertained that the intervention is tailored to meet the requirements of end-users and is viable for sustainable integration into clinical practice. This singular aspect distinguishes our eHealth tools from others, the overwhelming majority of which are conventionally conceived by software developers with minimal or no contributions from healthcare research teams (50). The technique could be compared to common linear development cycle models such as waterfall design so that design and potential usability problems could be identified early and corrected quickly, saving time and development costs. An iterative process of UCD was used to develop IVRS, which included personalized calls, feedback, and educational and tailoring calls. To our knowledge, there were no studies suggesting a UCD process for IVRS starting with need assessment.

### 4.2 Usability evaluation

We chose a pluralistic walkthrough as a popular usability technique to have experts evaluate the preliminary design. This method provided a range of skills and perspectives to focus on usability difficulties. However, this method makes it difficult to evaluate the performance of a task with any degree of accuracy. We assessed the following version using Thinking Aloud testing, a flexible and trustworthy evaluation technique. As a result, we received prompt and excellent user feedback.

The KT recipients stated one usability issue. To fix this, we updated the section to have fewer subsections, and we assigned code to each subsection that was linked to the section.

The usability testing of a system is one of the most essential characteristics for the adoption of IVRS, and other digital health systems (64). We developed an interface design that takes into account patient, needs and considers standards for good interface design. This interface must be considered in light of healthcare requirements based on the analysis of self-management behavior.

Out of the nine distinct usability challenges identified, two were uniformly encountered across all tasks: (1) the ambiguous functionality of the keys as indicated by their icons; and (3) the lack of assistance. In the ongoing investigation HCPs noted that the first and second obstacles were challenging and caused a significant consumption of their time. Our findings are consistent with those reported in prior research (65, 66). Consequently, it is imperative for designers and developers of health information systems to prioritize the enhancement of suboptimal navigation controls within these systems, as this concern is shared by both experts and users.

In the present evaluation of effectiveness, it was observed that the majority of HCPs successfully accomplished all three assigned tasks; however, two individuals encountered difficulties in completing tasks two and three. This inability may be attributed to the usability challenges we identified under the categories of “suboptimal search functionalities” and “inadequate data presentation and information management.” The issues related to “suboptimal search functionalities” and “inadequate data presentation” resulted in an extensive array of seizure conditions, thereby disorienting the participants. In a study aimed at investigating the usability of a physician-to-physician teleconsultation application within an orthopedic clinic, Choemprayong (2021) identified several usability concerns associated with mobile applications, including errors in data entry, challenges in presenting extensive datasets, and difficulties in item selection from lists (67). In his research, Chen emphasized the importance of effective navigation and search functionalities as critical determinants influencing users' evaluations of mobile health applications (68). Schwab (2018) contended that user-friendly navigation serves as the cornerstone of an exemplary mobile application, as it facilitates productivity and enhances overall effectiveness (69).

Based on the results of the usability test with end users (average SUS score of 81.2 and 88.8), we assume the IVRS is a user-friendly system. Currently, there are few research studies on user testing of IVRS: Thirumalai et al. (18) evaluated the usability of an IVRS aimed at increasing physical activity levels. This system received an average score of 81 (SD 5) on the SUS, they found challenges such as incentives for completing a call and incremental goal setting that were modifiable. Compared to this study, we encountered fewer usability issues and a similar score SUS. Korpershoek (41) studied the usability and usefulness of mobile self-management apps for COPD patients and found SUS to have a score of 91.

### 4.3 Limitations

A limitation of the study is that the IVRS was developed only for KT recipients and nurses responsible for patient education. Further development and usability testing for physicians remains to be done. One limitation of this study was that the usability sessions were not audio- or video-recorded, which would have allowed for deeper analysis of the loud comments. Operationalization is important phase of CeHRes roadmap, which unfortunately was not done in our study. It is suggested to use this section as the main section in studies. There are a number of obstacles such as IVRS software cost and lack of acceptance by a number of users or lack of motivation of users due to chronic disease. We overcame these obstacles by creating feedback along with a motivational message of the patient's behavior. On the other hand, there are facilitators such as the willingness of teaching nurses to use the IVRS for self-management, which reduces their workload and has changed education from face-to-face to virtual. Physicians also considered IVRS as useful and helpful for patients.

### 4.4 Recommendations

One of the notable strengths of this research lies in the systematic and comprehensive methodology employed in the development of IVRS aimed at supporting self-management, in alignment with the CeHRes roadmap and User-Centered Design (UCD), which actively involved key stakeholders across various forums, including focus groups, Delphi rounds, and surveys. This engagement is critical for obtaining profound insights into the specific requirements and potential obstacles related to the acceptance and utilization of technology. Furthermore, we utilized well-established theoretical frameworks, specifically Behavior Change Techniques (BCTs) and self-regulation theories, to guide the design and assessment of our IVRS, thereby enhancing both the reproducibility and efficacy of the intervention. For researchers and planners, this study can serve as a guide for developing IVRS interventions that meet end-user needs and preferences, have high potential for effectiveness, and can be used by the targeted community. In developing an IVRS, all phases are based on theory and evidence, and user needs and preferences are carefully considered. We have applied IVRS in a real-world setting and with real patients, which helps improve IVRS capabilities in an iterative process.

Future research should focus on evaluate the feasibility of IVRS interventions in daily practice in other chronic patients. The next step was to evaluate the impact of mobile Health (m-Health) interventions on meaningful patient outcomes and health care utilization. Recent studies of IVRS interventions in patients with KT recommend using Randomized Controlled Trials (RCTs) with sufficient sample size and 1-year follow-up to conclude about behavior change and treatment effects (41, 70).

## 5 Conclusion

The study discusses the design and usability testing of a self-management support system for KT recipients. The CeHRes roadmap employed to the development of an IVRS intervention tailored to the needs and preferences of the target populations, KT and HCP, with a high likelihood of improvement in medication adherence, knowledge and quality of life. An iterative process of UCD was used to develop IVRS, which included personalized calls, feedback, and educational and tailoring calls. Operationalization phase was not employed in our study. It is suggested to use this phase in studies. There are a number of obstacles such as IVRS software cost and lack of motivation of users due to chronic disease. We overcame these obstacles by creating feedback along with a motivational message of the patient's behaviour. This study rendered detailed reports about e-health intervention development. For researchers and planners, this study can serve as a guide for developing IVRS interventions that meet end-user needs and preferences, have high potential for effectiveness, and can be used by the targeted community.

## Data Availability

The datasets generated during and/or analyzed during the current study are available from the corresponding author on reasonable request.
